# Letter to the Editor: An ultra-sensitive assay using cell-free DNA fragmentomics for multi-cancer early detection

**DOI:** 10.1186/s12943-022-01594-w

**Published:** 2022-06-11

**Authors:** Hua Bao, Zheng Wang, Xiaoji Ma, Wei Guo, Xiangyu Zhang, Wanxiangfu Tang, Xin Chen, Xinyu Wang, Yikuan Chen, Shaobo Mo, Naixin Liang, Qianli Ma, Shuyu Wu, Xiuxiu Xu, Shuang Chang, Yulin Wei, Xian Zhang, Hairong Bao, Rui Liu, Shanshan Yang, Ya Jiang, Xue Wu, Yaqi Li, Long Zhang, Fengwei Tan, Qi Xue, Fangqi Liu, Sanjun Cai, Shugeng Gao, Junjie Peng, Jian Zhou, Yang Shao

**Affiliations:** 1 Geneseeq Research Institute, Nanjing Geneseeq Technology Inc., Nanjing, 210000 Jiangsu China; 2 Department of Liver Surgery and Transplantation, Liver Cancer Institute, Zhongshan Hospital, Fudan University, Shanghai, 200032 China; 3 Key Laboratory of Carcinogenesis and Cancer Invasion (Fudan University), Ministry of Education, Shanghai, 200032 China; 4 Shanghai Key Laboratory of Organ Transplantation, Zhongshan Hospital, Fudan University, Fudan University, 130 Fenglin Road, Shanghai, 200032 China; 5 Department of Colorectal Surgery, Fudan University Shanghai Cancer Center, Shanghai, 200032 China; 6 Department of Oncology, Shanghai Medical College, Fudan University, Shanghai, 200032 China; 7 Department of Thoracic Surgery, National Cancer Center/National Clinical Research Center for Cancer/Cancer Hospital, Chinese Academy of Medical Sciences and Peking Union Medical College, Beijing, 100730 China; 8 Key Laboratory of Minimally Invasive Therapy Research for Lung Cancer, Chinese Academy of Medical Sciences, Beijing, 100730 China; 9 Department of Thoracic Surgery, Peking Union Medical College Hospital, Chinese Academy of Medical Sciences, Beijing, 100730 China; 10 Department of Thoracic Surgery, China-Japan Friendship Hospital, Beijing, 100029 China; 11 Department of Cancer Institute, Fudan University Shanghai Cancer Center, Fudan University, Shanghai, 200032 China; 12 Institute of Biomedical Sciences, Fudan University, Shanghai, 200032 China; 13 State Key Laboratory of Genetic Engineering, Fudan University, Shanghai, 200032 China; 14 School of Public Health, Nanjing Medical University, Nanjing, 210029 Jiangsu China

**Keywords:** Multi-cancer early detection, Cell-free DNA, Fragmentomics, Machine learning

## Abstract

Early detection can benefit cancer patients with more effective treatments and better prognosis, but existing early screening tests are limited, especially for multi-cancer detection. This study investigated the most prevalent and lethal cancer types, including primary liver cancer (PLC), colorectal adenocarcinoma (CRC), and lung adenocarcinoma (LUAD). Leveraging the emerging cell-free DNA (cfDNA) fragmentomics, we developed a robust machine learning model for multi-cancer early detection. 1,214 participants, including 381 PLC, 298 CRC, 292 LUAD patients, and 243 healthy volunteers, were enrolled. The majority of patients (*N* = 971) were at early stages (stage 0, *N* = 34; stage I, *N* = 799). The participants were randomly divided into a training cohort and a test cohort in a 1:1 ratio while maintaining the ratio for the major histology subtypes. An ensemble stacked machine learning approach was developed using multiple plasma cfDNA fragmentomic features. The model was trained solely in the training cohort and then evaluated in the test cohort. Our model showed an Area Under the Curve (AUC) of 0.983 for differentiating cancer patients from healthy individuals. At 95.0% specificity, the sensitivity of detecting all cancer reached 95.5%, while 100%, 94.6%, and 90.4% for PLC, CRC, and LUAD, individually. The cancer origin model demonstrated an overall 93.1% accuracy for predicting cancer origin in the test cohort (97.4%, 94.3%, and 85.6% for PLC, CRC, and LUAD, respectively). Our model sensitivity is consistently high for early-stage and small-size tumors. Furthermore, its detection and origin classification power remained superior when reducing sequencing depth to 1× (cancer detection: ≥ 91.5% sensitivity at 95.0% specificity; cancer origin: ≥ 91.6% accuracy). In conclusion, we have incorporated plasma cfDNA fragmentomics into the ensemble stacked model and established an ultrasensitive assay for multi-cancer early detection, shedding light on developing cancer early screening in clinical practice.

**Supplementary Information:**

The online version contains supplementary material available at 10.1186/s12943-022-01594-w.

## Main text

The global cancer burden is increasing rapidly, and nearly 19.3 million new cases and 10.0 million cancer deaths were estimated in 2020 [1]. Over 60% of newly diagnosed cases and 70% of cancer mortality can be attributed to 10 common cancer types [1]. Among them, liver cancer, colorectal cancer, and lung cancer rank the top three causes and account for over one-third of cancer deaths [1]. Although cancer identified early is more likely to have a favorable prognosis [2], only limited early screening programs have been made available for specific cancer types [3]. Furthermore, detection limits, radiation exposure, fear of pain, monetary cost, etc., of existing screening programs are also obstacles in their implementation [4–6]. Therefore, exploring accurate and affordable biomarkers is needed for promoting early detection.

As a new class of biomarkers for cancer detection, cell-free DNA (cfDNA) in circulation is released from apoptosis and necrosis, and contains molecular signatures of its origin [7, 8]. For instance, tumor somatic mutations can serve as a classifier to distinguish circulating tumor DNA (ctDNA) shed from tumor cells and nontumorous cfDNA [9]. Epigenetic modifications such as DNA methylation and fragmentomic signatures such as fragmentation patterns and end motifs have also been utilized for identifying cancer [10–14]. However, assays based on single cfDNA features often yield inadequate detection ability, especially for stage I cases of prevalent cancer types [12, 14–16]. As identification at stage I often provides a better chance for the cure than later stages, developing more robust methods is critical to promote cancer early detection.

More recently, multi-dimensional predictive models that combine multiple fragmentomic and genomic features and even incorporate clinical information have improved their detection power for specific cancer types [12, 17]. Particularly, Ma et al. have leveraged the ensemble stacked strategy to integrate multiple fragmenomic features with machine learning algorithms and successfully built an ultrasensitive model for detecting stage 0/I colorectal adenocarcinoma [18]. Given the potential of the ensemble stacking strategy, we attempted to develop a multi-dimensional model using cfDNA fragmentomics from WGS data for multi-cancer detection and origin localization. Owing to their high prevalence and substantial impact, we built the model targeting liver, colorectal, and lung cancers in a cohort of the Chinese population. Our model demonstrated ultrasensitivity for cancer detection and accurately differentiated cancer origins, ideal for promoting cancer screening programs.

## Results and discussion

### Participant characteristics and disposition

**Fig. 1:**
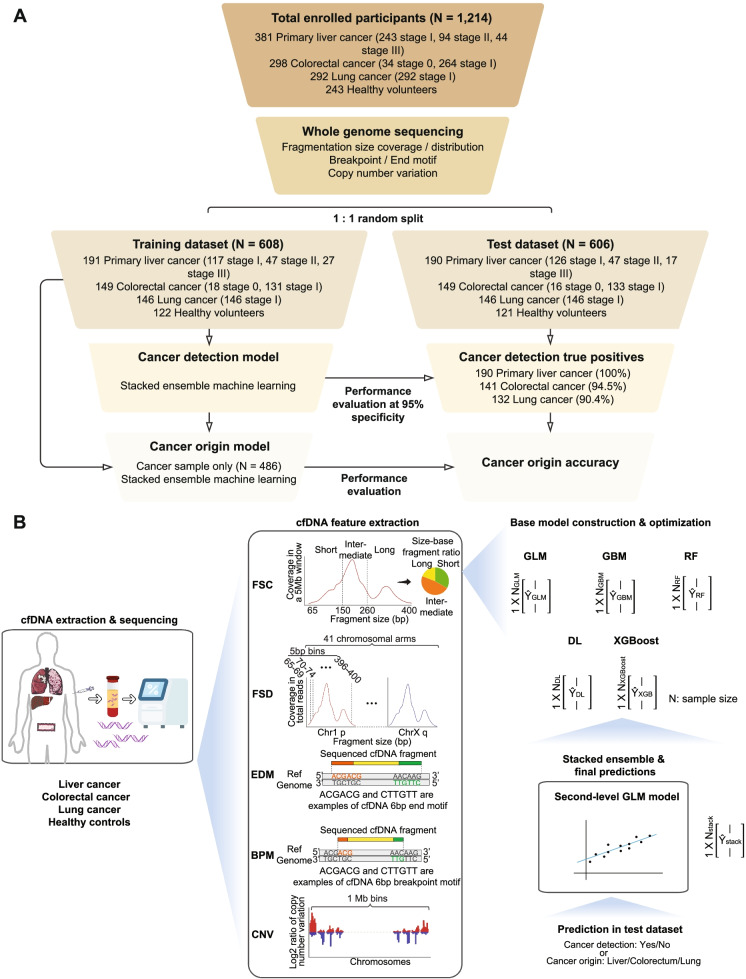
Schematic diagram of the study design. **A** The training cohort (*N* = 608) included 191 primary liver cancer (PLC), 149 colorectal cancer (CRC), 146 lung adenocarcinoma (LUAD) patients, and 122 healthy controls, which were used to train the cancer detection and cancer origin models. The test cohort (*N* = 606), which included 190 PLC, 149 CRC, 146 LUAD, and 121 healthy controls, was used to evaluate model performances. **B** Plasma samples were collected from PLC, CRC, LUAD patients, and healthy volunteers. The cfDNA was extracted from the participant's plasma sample and subject to whole-genome sequencing (WGS). Five different feature types, including Fragment Size Coverage (FSC), Fragment Size Distribution (FSD), EnD Motif (EDM), BreakPoint Motif (BPM), and Copy Number Variation (CNV), were calculated. For each feature type, a base model was constructed based on the ensemble learning of five algorithms- GLM, GBM, Random Forest, Deep Learning, and XGBoost. The base model predictions were then ensembled into a large matrix, subsequently used to train the final ensemble stacked model

We included 1,214 participants with previously untreated diseases: 381 primary liver cancer (PLC), 298 colorectal cancer (CRC), 292 lung adenocarcinoma (LUAD), and 243 healthy volunteers without cancer (Fig. 1A). This study was approved by the Ethnic Committees and in accordance with the ethical standards as laid down in the 1964 Declaration of Helsinki and its later amendments. Written informed consents were provided by all participants. Details about enrollment information are in Supplementary Materials and Methods. The participants were subject to WGS and fragmentomic feature extraction and randomly split into the training and test datasets in a 1:1 ratio. We took the whole training dataset to build the first-level cancer detection model and then the cancer samples in the training dataset to train the second-level cancer origin model. The workflow of model construction is described in Fig. 1B and Supplementary Materials and Methods. Briefly, we extracted five distinct features covering cfDNA fragmentation size, motif sequence, and copy number variation from the WGS data, namely Fragment Size Coverage (FSC), Fragment Size Distribution (FSD), EnD Motif (EDM), BreakPoint Motif (BPM), and Copy Number Variation (CNV). The fragmentomic features implemented five machine learning algorithms, including Generalized Linear Model (GLM), Gradient Boosting Machine (GBM), Random Forest, Deep Learning, and XGBoost, and integrated to establish the ensemble stacked model. It is worth noting that the model was built solely in the training dataset, while the test dataset remained untouched until the model was finalized. We evaluated the cancer detection model in the test dataset and then took the true-positive cases to validate the cancer origin model. Healthy and cancer participants' demographics and characteristics (Table S1) are comparable between the training and test datasets. More importantly, the cancer samples are highlighted by the majority of early-stage diseases [PLC: stage IA/IB 117/191 (61.3%) in the training cohort and 126/190 (66.3%) in test cohort; CRC: stage 0/I 149/149 (100.0%); LUAD stage IA/IB 146/146 (100.0%)].

### Differentiating cancer and non-cancer subjects by the cancer detection model

**Fig. 2:**
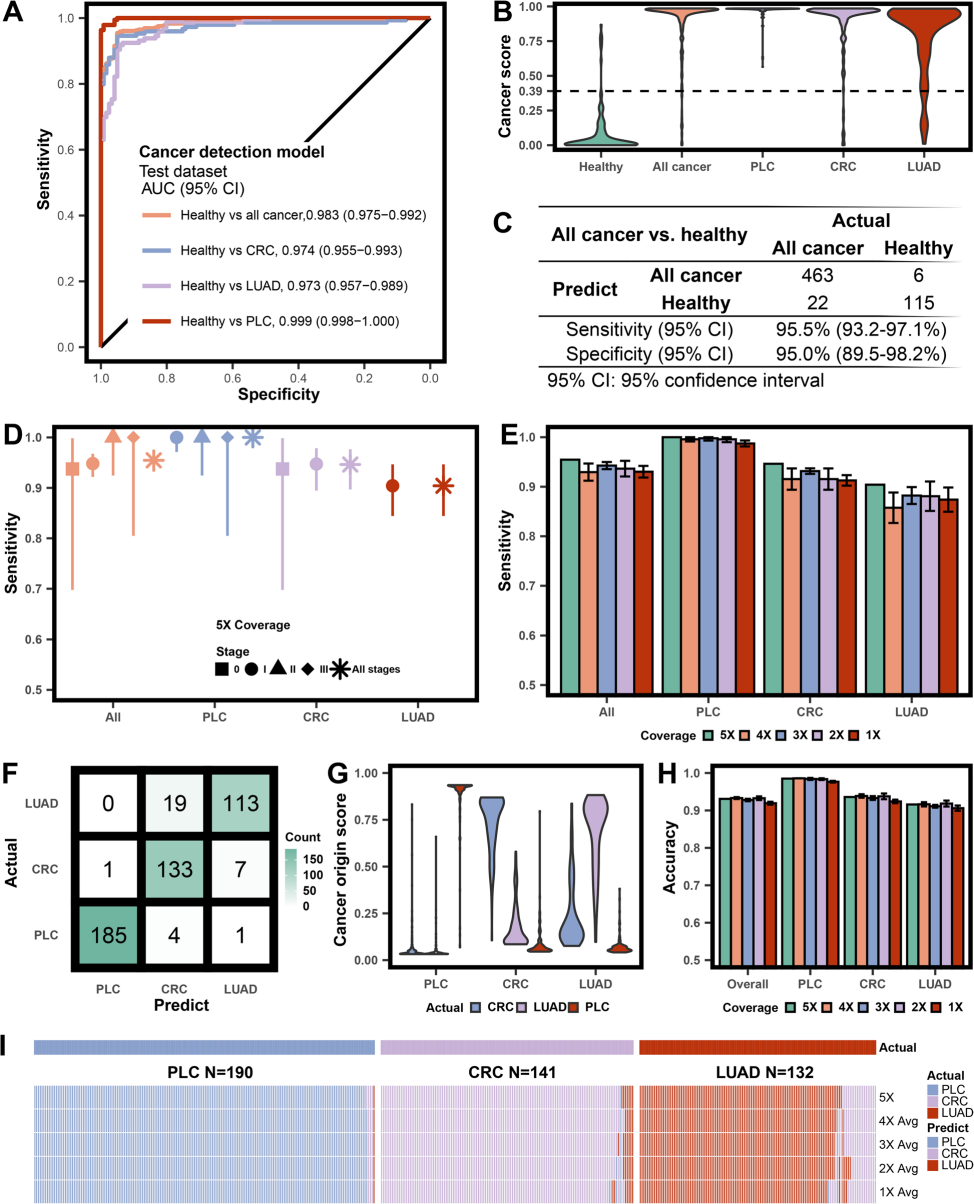
Performance and robustness valuation for the ensemble stacked model. **A** ROC curves evaluating the cancer detection model in distinguishing cancer patients from healthy volunteers in the test cohort, and further categorized into each cancer type class. **B** Violin plots illustrating cancer score distribution in the healthy, all cancer, primary liver cancer (PLC), colorectal cancer (CRC), and lung adenocarcinoma (LUAD) groups in the test cohort predicted by the cancer detection model. The 95% specificity cutoff for cancer score was 0.39, as shown by the dotted line. **C** Performance of the cancer detection model in identifying all cancer patients. **D** Dot plot of sensitivity in cancer detection by each cancer type and/or stage, at 95% specificity. The error bars represented the 95% confidence interval. **E** Robustness test for the cancer detection model using test cohort with downsampled coverage depth (4×-1×). The error bars were calculated based on five repeats for each coverage. **F** Confusion matrix of the selected test cohort by cancer detection model for the cancer origin model. **G** Violin illustrating cancer origin score distribution in the PLC, CRC, and LUAD groups in the selected test cohorts predicted by the cancer origin model. **H** Dot plot illustrating robustness test for the cancer origin model using the selected test cohort with downsampled coverage depth (4×-1×). **I** Heatmap illustrating the detailed results of each patient for the robustness test of the cancer origin model

We reached a superior AUC value of 0.983 (95% CI: 0.975-0.992) for detecting all cancer subjects in the test dataset (Fig. 2A). The PLC group has the highest AUC (0.999, 95% CI: 0.975-0.992), followed by the CRC (0.974, 95% CI: 0.955-0.993) and LUAD (0.973, 95% CI: 0.957-0.989) groups. Healthy subjects have lower cancer scores than cancer subjects, and the three cancer types showed similar score distribution (Fig. 2B). The cancer score of 0.39 rendered a 95.0% specificity (95% CI: 89.5-98.2%). The corresponding sensitivities are 95.5% (95% CI: 93.2-97.1%) for all cancer subjects (Fig. 2C), and 100.0% (95% CI: 98.1-100.0%), 94.6% (95% CI: 89.7-97.7%), and 90.4% (95% CI: 84.4-94.7%) for PLC, CRC and LUAD, respectively (Table S2). We observed an upward trend from the early to later stages for the distribution of cancer scores in all-cancer, PLC, and CRC classes (Fig. S1). A propensity score matching analysis balanced the age and sex factors between cancer and non-cancer groups in the test dataset. The resultant subset consisting of 113 PLC, 73 CRC, 85 LUAD, and 85 age and gender-matched healthy controls remained high performance in distinguishing cancer patients from non-cancer controls (AUC: 0.988, 95% CI: 0.980-0.996, Fig. S2A). We also performed 10-fold cross-validation during training to evaluate model overfitting. The 10-fold cross-validation AUCs for all-cancer and individual cancer types were equally high compared to the independent test dataset (Fig. S2B), reassuring that overfitting was not a major concern.

Our model exhibited ultrasensitivity in detecting cancers at various stages (Fig. 2D). The sensitivity is above 90% for stages 0 and I, and elevated to nearly 100% for stages II and III. Furthermore, we used patient demographics and clinical characteristics to categorize disease subgroups for evaluation (Table S3 and Figs. S3-S5). The model's detection sensitivity was consistently high even in the challenging categories, such as MIA and <1 cm tumors of LUAD. We assessed the model's robustness by gradually down-sampling the coverage to 1× (Fig. 2E Table S4). Despite a slight dip, the model remained stable with over 91.5% sensitivity for all-cancer. Even for the least detectable class of LUAD, the sensitivity at 1× is still above 87%.

Furthermore, the cancer detection model was assessed in a preliminary at-risk patient cohort and showed an overall specificity of 92.4% (Table S5, details in Supplementary Results).

### Locating cancer at its origin by the cancer origin model

All test dataset patients correctly identified as "Cancer" by the cancer detection model were subsequently analyzed in the cancer origin model. The model correctly identified the cancer origin for 431 patients (accuracy 0.931, 95% CI: 0.900-0.950) for the three cancer types (Fig. 2F and Table S6). The sensitivities for individual cancer types were 97.4% (95% CI: 94.0-99.1%), 94.3% (95% CI: 89.1-97.5%), and 85.6% (95% CI: 78.4-91.1%) for PLC, CRC, and LUAD, respectively. We plotted the cancer origin scores of each type for all patients (Fig. 2G). Generally, the top scores matched the true cancer types. Such consistency is the most compelling for the PLC patients, followed by the CRC patients, while the LUAD group has more erroneous CRC predictions (Fig. 2F and G). We further inspected the origin scores of the misinterpreted patients (Fig. S6). The score differences between the true origin and the misinterpreted class were minimal (≤ 0.05) for potential improvement. The cancer origin model is robust with lower coverage WGS data (Fig. 2H and Table S7). The accuracies for PLC, CRC, and LUAD at 1× coverage are 97.7%, 92.4%, and 90.6%, respectively, whereas the predictions of each patient at different sequencing coverages were listed in Fig. 2I, H.

Our study has several limitations. First, we performed the proof-of-concept study using liver cancer, colorectal cancer, and lung cancer for their high prevalence. Targeting a broader population and more cancer types, including the less prevalent ones, would be necessary to develop the assay and eliminate cancer treatment inequity. Second, we are expanding our current cohort size to enable independent validation and improve the estimation accuracy of relatively small-size subgroups (e.g., cHCC-ICC, MIA, stage IB LUAD).

## Conclusions

By integrating multiple fragmentomic features from cfDNA WGS data, our ensemble stacked model exhibited superior detection and localization power for the prevalent cancer types of PLC, CRC, and LUAD even at stages 0 and I. The robustness of our model is consistently high using as low as 1× sequencing coverage depth, suitable for developing accurate and affordable early detection assays for clinical practice.

## Supplementary Information

**Additional file 1 : Supplementary Table 1**. Participant demographics and baseline characteristics. **Supplementary Table 2**. Performance of the first-level cancer detection model. **Supplementary Table 3**. Performances of the cancer detection model on different subgroups based on clinical characteristics. **Supplementary Table 4**. Cancer detection model robustness test using downsampled (4× to 1× coverage depths) WGS data. Each downsampled coverage depth was repeated five times. **Supplementary Table 5**. Performances of the cancer detection model on extra healthy volunteer and at-risk patient cohort. **Supplementary Table 6**. The performance of the second level cancer origin model shown in the confusion matrix table. **Supplementary Table 7**. Cancer origin model robustness test using downsampled (4× to 1× coverage depths) WGS data. Each downsampled coverage depth was repeated five times.

**Additional file 2: Supplementary Materials and Methods. Supplementary Figure 1**. Distribution of cancer scores by cancer stages. **Supplementary Figure 2**. Cancer detection model evaluation using matched test cohort and cross-validated training cohort. **Supplementary Figure 3**. Evaluating cancer detection model sensitivity within different primary liver cancer subgroups. **Supplementary Figure 4**. Evaluating cancer detection model sensitivity within different colorectal cancer subgroups. **Supplementary Figure 5**. Evaluating cancer detection model sensitivity within different lung adenocarcinoma subgroups. **Supplementary Figure 6**. Cancer origin scores of the false-negative samples.

## Data Availability

The data that support the findings of this study are available from the corresponding authors upon reasonable request.

## Abbreviations

AUC: area under the curve
cfDNA: cell-free DNA
BPM: breakpoint motif
CI: confidence interval
CNV: copy number variation
ctDNA: circulating tumor DNA
CRC: colorectal adenocarcinoma
EDM: end motif
FSD: fragment size distribution
FSC: fragment size coverage
GBM: gradient boosting machine
GLM: generalized linear model
HCC: hepatocellular carcinoma
ICC: intrahepatic cholangiocarcinoma
LUAD: lung adenocarcinoma
MIA: minimally invasive adenocarcinoma
PLC: primary liver cancer
ROC: receiver operating characteristic
WGS: whole-genome sequencing
