# Supplementary material for: Letter to the Editor: An ultra-sensitive assay using cell-free DNA fragmentomics for multi-cancer early detection
Source: Mol Cancer. 2022 Jun 11;21:129. doi: 10.1186/s12943-022-01594-w (PMC9188251; doi:10.1186/s12943-022-01594-w)

**Title: An ultra-sensitive assay using cell-free DNA fragmentomics for multi-cancer early detection**

**Supplementary Materials and Methods**

Early detection offers an effective means to beat cancer, as cancer identified early is more likely to respond to less expensive and morbid treatments, resulting in a favorable prognosis to patients [[1](#_ENREF_1)]. To date, only several early screening options have been made available for patients with specific cancer types [[2](#_ENREF_2)]. For example, commonly used methods for early detection of lung cancer, colorectal cancer, and liver cancer include low-dose computed tomography (LDCT), Stool Blood Test (SBT), and Optical Colonoscopy (OC), as well as serum Alpha-fetoprotein (AFP) and ultrasound-based surveillance, respectively. However, detection limits, radiation exposure, fear of pain, monetary cost, etc., have posed obstacles to implementing the early detection tests in clinical practice [[3-6](#_ENREF_3)]. Recently, epigenetic modifications such as DNA methylation and ctDNA fragmentomic signatures such as fragmentation patterns and end motifs have shown great potential for early cancer detection [[7-11](#_ENREF_7)]. Multi-dimensional predictive models can increase detection power by integrating multiple fragmentomic and genomic features. A model combining cfDNA methylation, fragmentation, end motif, and nucleosome footprint patterns reached sensitivity over 95% for hepatocellular carcinoma (HCC) prediction and demonstrated higher performance than single feature-based models [[12](#_ENREF_12)]. Analyzing clinical risk factors, protein biomarker levels, medical images, and cfDNA fragment size patterns in one model further promoted the model’s performance for detecting cancer [[10](#_ENREF_10)]. However, it is conceivable that the assay complexity could introduce the monetary and time cost. Leveraging the power of ensemble stacked machine learning model, Ma et al. simplied the assay on the basis of whole-genome sequencing (WGS) alone and created an ultrasensitive model using five cfDNA fragmentomic features for stage 0/I colorectal adenocarcinoma detection, likewise showing that the ensemble stacked approach out-performed any single algorithm/feature type. [[13](#_ENREF_13)].

In this study, we aimed to establish an integrated machine learning model using cfDNA fragmentomics from WGS data for robust detection and origin localization of multiple cancer types using a cohort of the Chinese population.

***Participant enrollment***

The primary liver cancer (PLC) patients, including hepatocellular carcinoma (HCC), intrahepatic cholangiocarcinoma (ICC), and c(combined)HCC-ICC, the colorectal cancer (CRC) patients including stage 0 (carcinoma *in situ*) and stage I, and the lung adenocarcinoma (LUAD) patients (all in stage I) were diagnosed with the American Joint Committee on Cancer (AJCC) TNM system (8^th^ edition) [[14](#_ENREF_14)]. Healthy volunteers had no history of cancer diagnosis/treatment and no cancer-related symptoms within 30 days prior to enrollment. Individuals excluded from this study include participants during pregnancy or lactation, participants of ongoing fever or recipient of anti-inflammation therapy within 14 days prior to study blood draw, recipients of blood transfusion within 30 days prior to study blood draw, and recipients of organ transplant or prior non-autologous (allogeneic) bone marrow or stem cell transplant. The PLC patients and healthy volunteers were enrolled from Liver Cancer Institute, Zhongshan Hospital, Fudan University. The CRC patients were enrolled from the Department of Colorectal Surgery, Shanghai Cancer Center, Fudan University. The LUAD patients were from 3 different medical centers (Cancer Hospital, Chinese Academy of Medical Sciences, Peking Union Medical College Hospital, and China-Japan Friendship Hospital). This study was approved by the ethics committee of each participating hospital and in accordance with international standards of good clinical practice. Written informed consents were provided by all participants.

***cfDNA sequencing and bioinformatics pipelines***

Blood samples collected from participants underwent cell-free DNA (cfDNA) extraction followed by WGS. We performed sample collection, cfDNA extraction, and whole-genome sequencing (WGS) uniformly as follows: The venous blood samples were collected during routine physical checks (healthy volunteers) or pre-surgery (cancer patients) into EDTA blood collection tubes (Becton Dickinson). Samples were kept at 4℃ for no more than 2 hours prior to centrifugation. Blood samples were centrifuged at 1,800 g for 10 mins at 4℃ for plasma collection. Plasma samples were centrifuged a second time at 16,000 g for 10 mins at 4℃ to remove cell debris. Then samples were frozen, shipped to the Clinical Laboratory Improvement Amendments (CLIA)- certified and College of American Pathologists (CAP)- accredited clinical testing laboratory (Nanjing Geneseeq Technology Inc., China) on dry ice, and stored at -80℃ to for processing together. The extraction of cfDNA was performed automatically on Hamilton Microlab STAR automated liquid handling platform (Hamilton Company). The cfDNA was extracted using QIAamp Circulating Nucleic Acid Kit (Qiagen), and the concentration was measured with Qubit dsDNA HS Assay Kit (Thermo Fisher Scientific). 5-10 ng of cfDNA per sample was subject to PCR-free WGS library construction using the KAPA Hyper Prep Kit (KAPA Biosystems). The library was constructed automatically on Biomek (Beckman Coulter), quantified using the KAPA SYBR FAST qPCR Master Mix (KAPA Biosystems), and underwent paired-end sequencing on NovaSeq platforms (Illumina).

For bioinformatic analysis, raw sequencing data were first trimmed by Trimmomatic as part of the quality control (QC) protocol [[15](#_ENREF_15)]. The qualified reads were then mapped onto the human reference genome (GRCh37/UCSC hg19) using the sequence aligner BWA [[16](#_ENREF_16)] after PCR duplicates removal by Picard toolkit (http://broadinstitute.github.io/picard/).

***Fragmentomic feature identification***

The overall modeling workflow is demonstrated in Fig. 1B. From the processed WGS data, we extracted five fragmentomic features covering cfDNA fragmentation size, motif sequence, and copy number variation. Fragmentomic feature profiles collected from the training dataset were used to construct multi-dimensional machine learning models for both cancer detection and localization. In contrast, the test dataset remained untouched during the model construction and was solely used to evaluate the model performance afterward.

Two fragment size profiles, Fragment Size Coverage (FSC) and Fragment Size Distribution (FSD), were optimized from published fragment size study and have shown improvement of the prediction ability as demonstrated in our previous study [[8](#_ENREF_8), [13](#_ENREF_13)]. The fragment sizes were used to construct fragmentation profiles with in-house scripts. The FSC was adapted from the DELFI method [[8](#_ENREF_8)] and optimized by introducing an extra fragment size group and using improved cutoff. It was generated using the coverages of short (65-150bp), intermediate (151-260bp), long (261-400bp), and total (65-400bp) cfDNA fragments. The extended ranges allowed the inclusion of broader size regions than what DELFI has reported. The genome was firstly divided into 100 kB bins. Next, the coverage of the four fragment size groups in each 100 kB bin was calculated and corrected by GC content. We then combined the coverages in every 50 contiguous 100 kB bins to calculate the coverage in the corresponding 5 MB (50 × 100 kB) bin. For each fragmentation size group, the scaled coverage score (z-score) in every 5 MB bin was calculated by comparing the variable value against the overall mean value. The FSD feature examined the coverage of cfDNA fragments ranging from 65 bps to 400 bps in 5 bp stepwise (e.g., 65-69 bps, 70-74 bps…) at every chromosome arm. The raw coverage score of FSD was also scaled into the z-score by comparing the variable value against the overall mean value. The FSC and FSD z-scores were subsequently used by the machine learning algorithms.

After aligning the sequencing reads to the human reference genome hg19, we obtained the features of motif profiles. Two types of motifs, the EnD Motif (EDM) and BreakPoint Motif (BPM), were extracted using in-house scripts as previously described [[13](#_ENREF_13)]. The EDM was adapted from the 4bp end motif as initially reported by Jiang et al. and extended to 6bp to increase the diversity [[17](#_ENREF_17)]. We then calculated the frequency of each 6bp end motif pattern. The sum of all EDM frequencies is equal to 1. The BPM profiled the 3bp genomic DNA sequences upstream and downstream of cfDNA 5’ end breakpoints and examined the frequency of different 6bp motifs in the human genome. The sum of all BPM frequencies is also equal to 1. The EDM and BPM features can classify samples based on the sequences at the end of cfDNA fragments.

The Copy Number Variation (CNV) profile was calculated using ichorCNA as reported by Wan et al. [[18](#_ENREF_18)]. First, the genome of each sample was divided into 1 MB bins. For each bin, the depth after bin-level GC correction was used by a Hidden Markov Model (HMM) to compare against the software baseline. Then, we calculated the log_2_ ratio for the CNV score.

***Machine learning model construction***

For the binary classification cancer detection model, five base models were constructed using FSC, FSD, EDM, BPM, and CNV based on the training dataset only (Fig. 1B), while the test dataset remained locked until performance evaluation. Each one of the five base models implemented five types of algorithms, including Generalized Linear Model (GLM), Gradient Boosting Machine (GBM), Random Forest (RF), Deep Learning (DL), and XGBoost. Healthy control and cancer patient samples from the training dataset were used to train the given algorithm, which generated the corresponding cancer score ranging from 0 to 1 for every sample. A higher score output by the models represented a higher probability for cancer. The cancer scores from all five algorithms were ensembled into a matrix, which was subsequently used by a second layer GLM algorithm to create the corresponding base model (Fig. 1). Ten-fold cross-validation was performed for each of these five base models for parameter optimization. The optimal base models were then used to create the final multi-dimensional model through ensemble stacked machine learning. The cancer scores predicted by each of the five base models were ensembled into a matrix as input for the ensemble stack algorithm to generate the final cancer detection model. After that, the untouched test cohort was used to evaluate the detection performance of the multi-dimensional ensembled machine learning model at 95% specificity.

The cancer origin model was built using the cancer samples from the training dataset. Similar to the cancer detection model, an ensemble stacked machine learning model was constructed based on five base models using the FSC, FSD, EDM, BPM, and CNV features, with each base model employing the five algorithms above. In the cancer origin model, three cancer origin possibility scores (ranging from 0 to 1) of the liver, colorectum, and lung were calculated for each patient, while the total value of these three scores for each subject is 1.0. Unlike the binary cancer detection model, the cancer origin model runs multi-class classification, and the category with the highest score among the three cancer types was predicted as the cancer origin. The true-positive predictions from the test dataset by the cancer detection model were used to evaluate the cancer origin model.

***Statistical Analysis***

The receiver operating characteristic (ROC) curves were constructed using the pROC package (v. 1.17.0.1). Based on true-positive (TP), true-negative (TN), false-positive (FP), and false-negative (FN) of cancer prediction, the sensitivity [TP/(TP+FN)], specificity [TN/(TN+FP)], positive predictive value (PPV) [TP/(TP+FP)] and negative predictive value (NPV) [TN/(TN+FN)] values, accuracy [(TP+TN)/(TP+FP+TN+FN)] as well as the corresponding 95% confidence intervals, were calculated using the epiR package (v 2.0.19). Propensity score matching analysis of age and gender within the test cohort was performed using the MatchIt package (4 4.2.0). All statistical analyses were performed in R (v.3.6.3).

**Supplementary Results**

***Evaluating model performance in at-risk patient cohort***

We have tested the cancer detection model in an external cohort containing at-risk patients with benign diseases, using preliminary data being collected for another study. The cohort enrolled 175 participants who were over 45 years of age, including 75 healthy participants and 100 patients with benign diseases such as bengin nodules or prostatic hyperplasia, from multiple physical examination centers. A total of 3 samples were excluded from the cohort due to quality control. Our model was able to achieve an overall specificity of 92.4% (95% CI: 87.4-95.9%). As shown in Table S5, the specificity was slightly higher in the healthy participants (94.5%, 95% CI: 86.6-98.5%) compared to patients with benign diseases (90.9% 83.4-95.8%).

**Supplementary References:**

1. World Health Organization: **Guide to cancer early diagnosis.** World Health Organization; 2017.

2. Chen X, Gole J, Gore A, He Q, Lu M, Min J, Yuan Z, Yang X, Jiang Y, Zhang T, et al: **Non-invasive early detection of cancer four years before conventional diagnosis using a blood test.** *Nat Commun* 2020, **11:**3475.

3. Patel M, Shariff MI, Ladep NG, Thillainayagam AV, Thomas HC, Khan SA, Taylor-Robinson SD: **Hepatocellular carcinoma: diagnostics and screening.** *J Eval Clin Pract* 2012, **18:**335-342.

4. National Lung Screening Trial Research T, Church TR, Black WC, Aberle DR, Berg CD, Clingan KL, Duan F, Fagerstrom RM, Gareen IF, Gierada DS, et al: **Results of initial low-dose computed tomographic screening for lung cancer.** *N Engl J Med* 2013, **368:**1980-1991.

5. Daskalakis C, DiCarlo M, Hegarty S, Gudur A, Vernon SW, Myers RE: **Predictors of overall and test-specific colorectal Cancer screening adherence.** *Prev Med* 2020, **133:**106022.

6. Parikh ND, Mehta AS, Singal AG, Block T, Marrero JA, Lok AS: **Biomarkers for the Early Detection of Hepatocellular Carcinoma.** *Cancer Epidemiol Biomarkers Prev* 2020, **29:**2495-2503.

7. Lo YMD, Han DSC, Jiang P, Chiu RWK: **Epigenetics, fragmentomics, and topology of cell-free DNA in liquid biopsies.** *Science* 2021, **372**.

8. Cristiano S, Leal A, Phallen J, Fiksel J, Adleff V, Bruhm DC, Jensen SO, Medina JE, Hruban C, White JR, et al: **Genome-wide cell-free DNA fragmentation in patients with cancer.** *Nature* 2019, **570:**385-389.

9. Liu MC, Oxnard GR, Klein EA, Swanton C, Seiden MV, Consortium C: **Sensitive and specific multi-cancer detection and localization using methylation signatures in cell-free DNA.** *Ann Oncol* 2020, **31:**745-759.

10. Mathios D, Johansen JS, Cristiano S, Medina JE, Phallen J, Larsen KR, Bruhm DC, Niknafs N, Ferreira L, Adleff V, et al: **Detection and characterization of lung cancer using cell-free DNA fragmentomes.** *Nat Commun* 2021, **12:**5060.

11. Liu J, Zhao H, Huang Y, Xu S, Zhou Y, Zhang W, Li J, Ming Y, Wang X, Zhao S, et al: **Genome-wide cell-free DNA methylation analyses improve accuracy of non-invasive diagnostic imaging for early-stage breast cancer.** *Mol Cancer* 2021, **20:**36.

12. Chen L, Abou-Alfa GK, Zheng B, Liu JF, Bai J, Du LT, Qian YS, Fan R, Liu XL, Wu L, et al: **Genome-scale profiling of circulating cell-free DNA signatures for early detection of hepatocellular carcinoma in cirrhotic patients.** *Cell Res* 2021, **31:**589-592.

13. Ma X, Chen Y, Tang W, Bao H, Mo S, Liu R, Wu S, Bao H, Li Y, Zhang L, et al: **Multi-dimensional fragmentomic assay for ultrasensitive early detection of colorectal advanced adenoma and adenocarcinoma.** *J Hematol Oncol* 2021, **14:**175.

14. Amin MB, Greene FL, Edge SB, Compton CC, Gershenwald JE, Brookland RK, Meyer L, Gress DM, Byrd DR, Winchester DP: **The Eighth Edition AJCC Cancer Staging Manual: Continuing to build a bridge from a population-based to a more "personalized" approach to cancer staging.** *CA Cancer J Clin* 2017, **67:**93-99.

15. Bolger AM, Lohse M, Usadel B: **Trimmomatic: a flexible trimmer for Illumina sequence data.** *Bioinformatics* 2014, **30:**2114-2120.

16. Li H, Durbin R: **Fast and accurate short read alignment with Burrows-Wheeler transform.** *Bioinformatics* 2009, **25:**1754-1760.

17. Jiang P, Sun K, Peng W, Cheng SH, Ni M, Yeung PC, Heung MMS, Xie T, Shang H, Zhou Z, et al: **Plasma DNA End-Motif Profiling as a Fragmentomic Marker in Cancer, Pregnancy, and Transplantation.** *Cancer Discov* 2020, **10:**664-673.

18. Wan N, Weinberg D, Liu TY, Niehaus K, Ariazi EA, Delubac D, Kannan A, White B, Bailey M, Bertin M, et al: **Machine learning enables detection of early-stage colorectal cancer by whole-genome sequencing of plasma cell-free DNA.** *BMC Cancer* 2019, **19:**832.

**Supplementary Figures**

**Supplementary Figure 1. Distribution of cancer scores by cancer stages.** Violin plots illustrating cancer score distribution in the healthy, all cancer, primary liver cancer (PLC), colorectal cancer (CRC), and lung adenocarcinoma (LUAD) groups by the disease stages in the test cohort. The 95% specificity cutoff for cancer score was 0.39, as shown by the dotted line. The triangle marks indicate the mean values of each group.


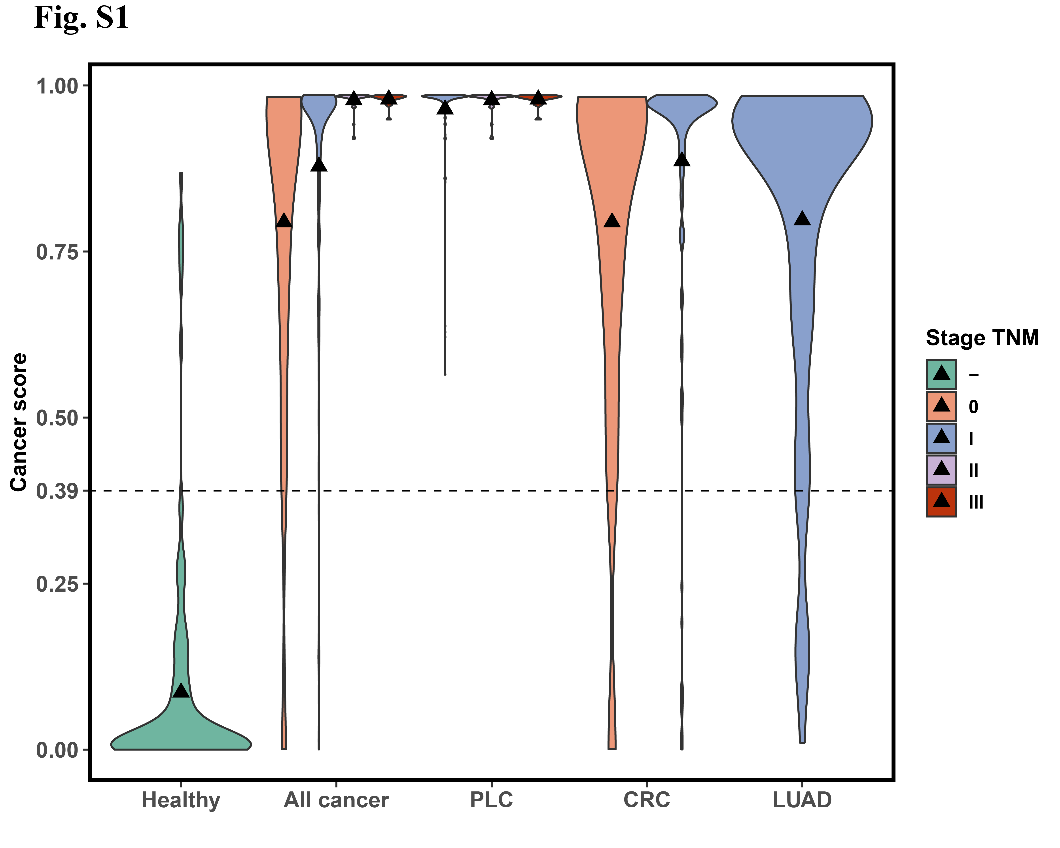


**Supplementary Figure 2. Cancer detection model evaluation using matched test cohort and cross-validated training cohort. A** ROC curves generated using an age and gender-matched subset of the test cohort. The cancer detection model was evaluated for distinguishing cancer patients (all cancer and specific cancer classes) from healthy volunteers. **B** ROC curves generated using 10-fold cross-validation results on the training cohort were used for evaluating the cancer detection model in distinguishing cancer patients from healthy volunteers and specific cancer classes. PLC: Primary liver cancer; CRC: Colorectal cancer; LUAD: lung adenocarcinoma.


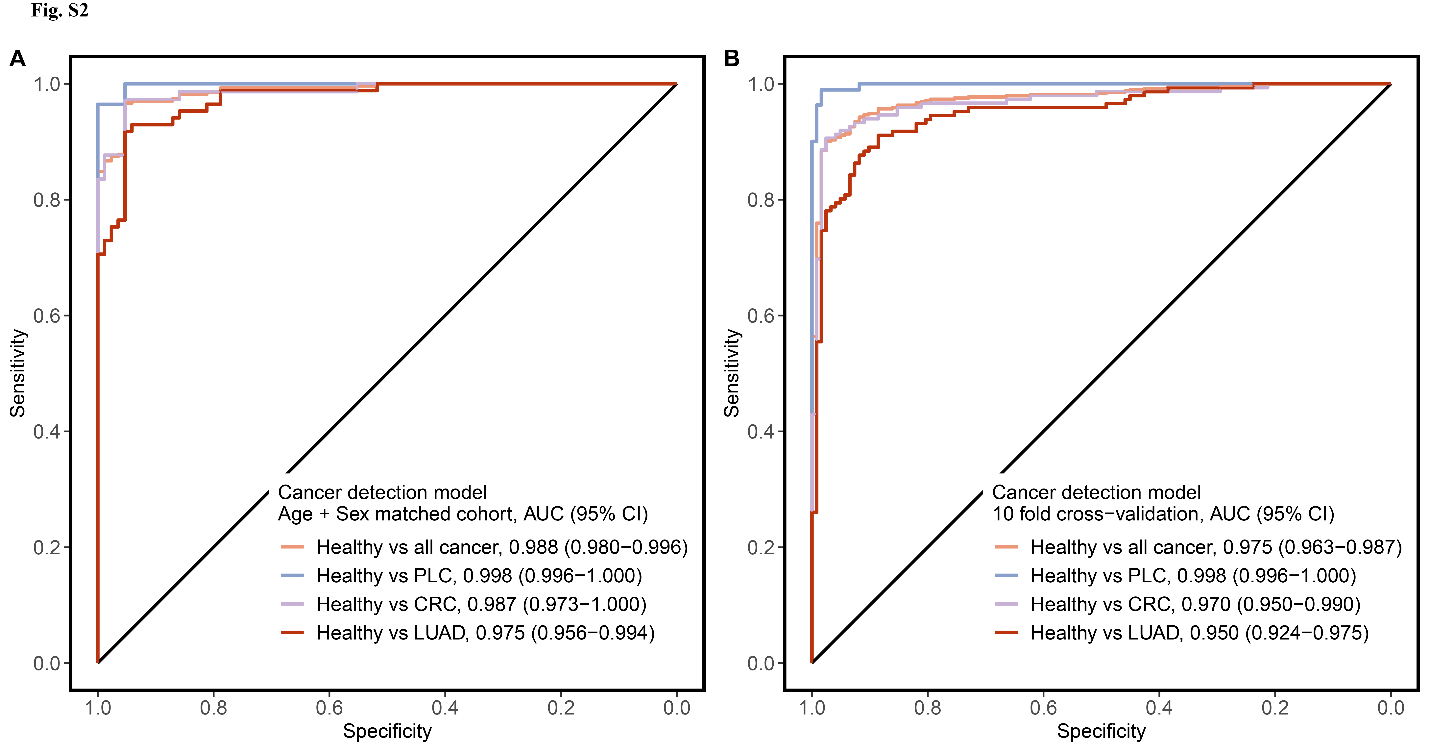


**Supplementary Figure 3. Evaluating cancer detection model sensitivity within different primary liver cancer subgroups.** Error bars represent 95% confidence intervals. HCC: hepatocellular carcinoma; ICC: intrahepatic cholangiocarcinoma; LC: Liver cirrhosis; ALB: Albumin; AFP: Alpha-fetoprotein; TBIL: Total bilirubin level.


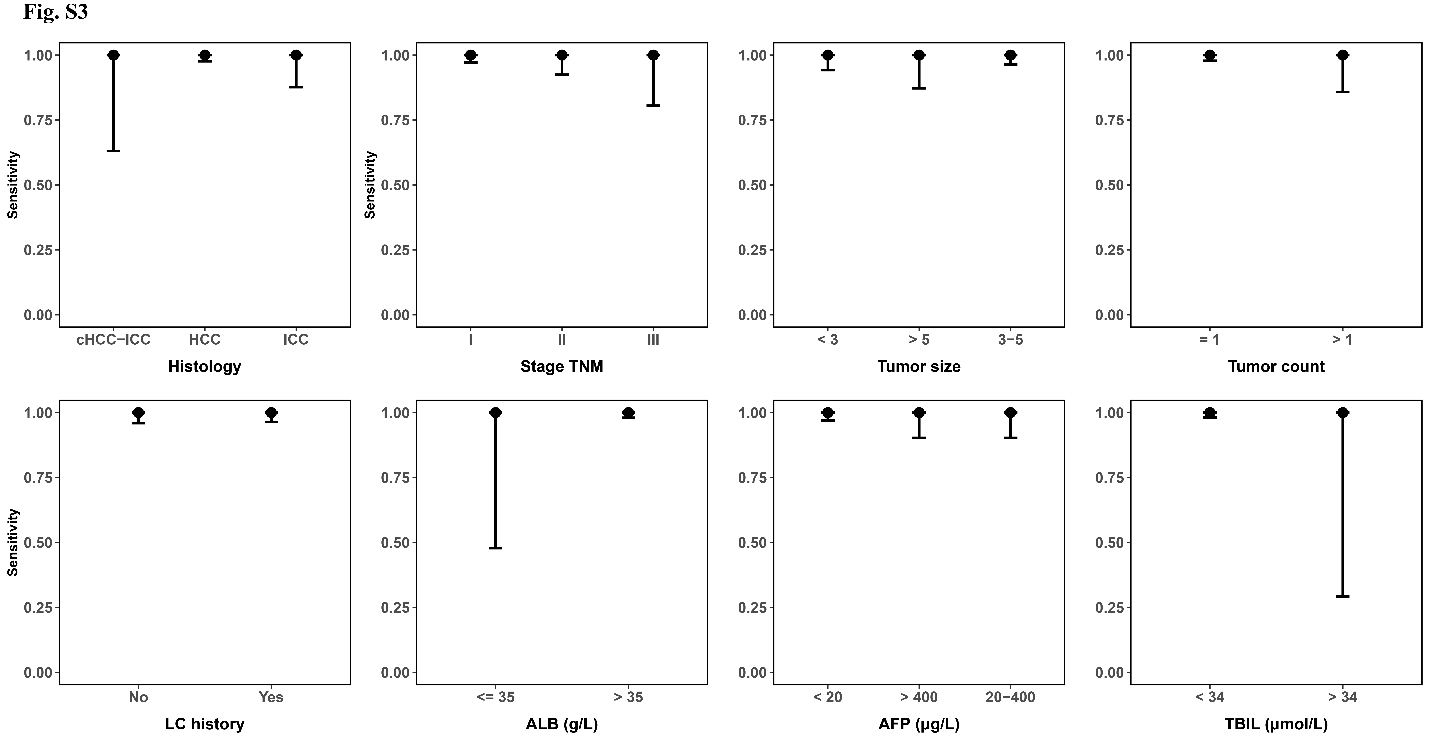


**Supplementary Figure 4. Evaluating cancer detection model sensitivity within different colorectal cancer subgroups.** Error bars represent 95% confidence intervals. Neg: Negative; Pos: Positive; FOBT: Fecal occult blood test; CA199: Carbohydrate antigen 19-9; CEA: Carcinoembryonic antigen; Mut: Mutated; WT: Wild type; dMMR: deficient mismatch repair; pMMR: proficient mismatch repair.


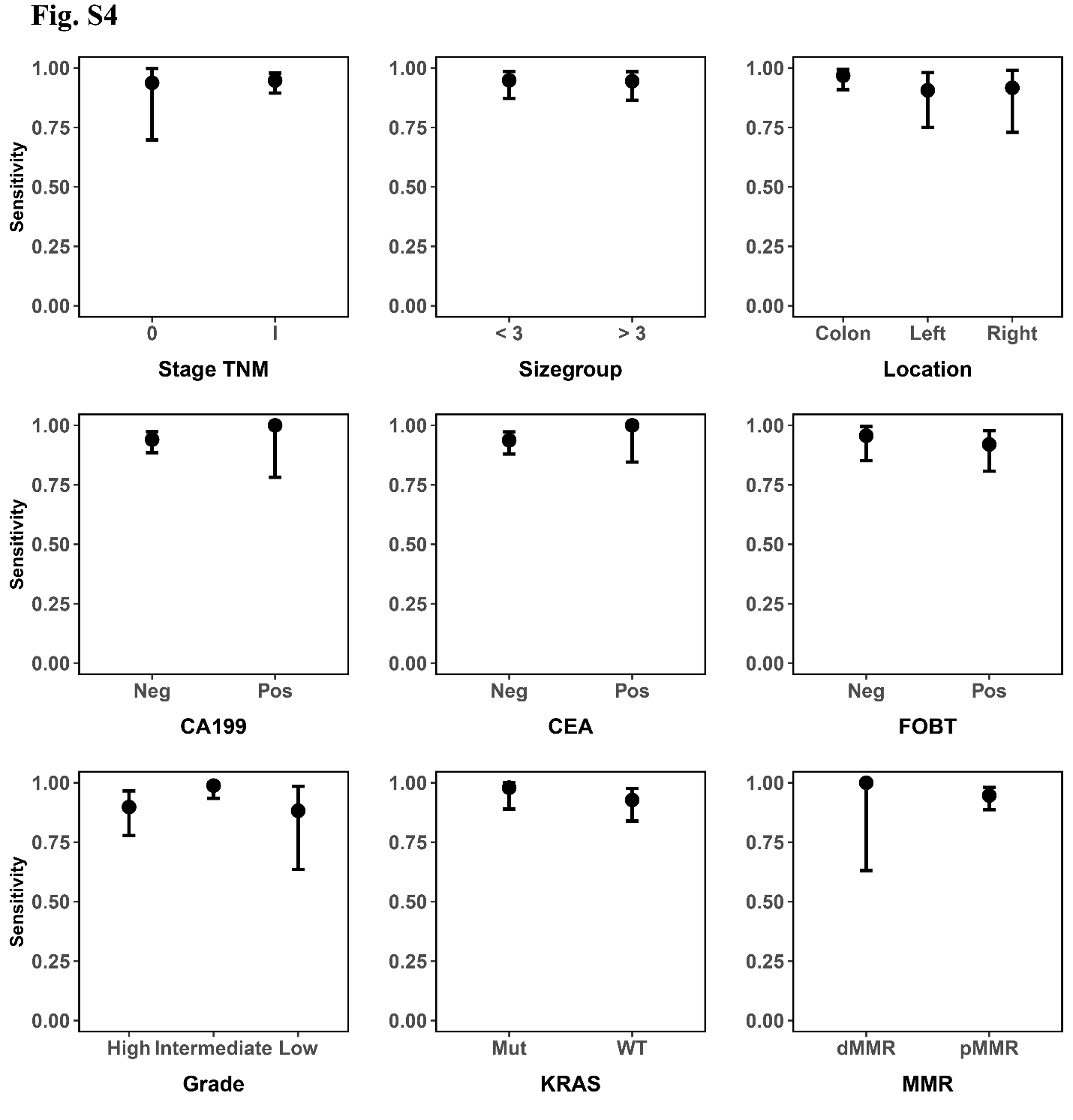


**Supplementary Figure 5. Evaluating cancer detection model sensitivity within different lung adenocarcinoma subgroups.** Error bars represent 95% confidence intervals. ADC: Adenocarcinoma; MIA: Minimally invasive adenocarcinoma.


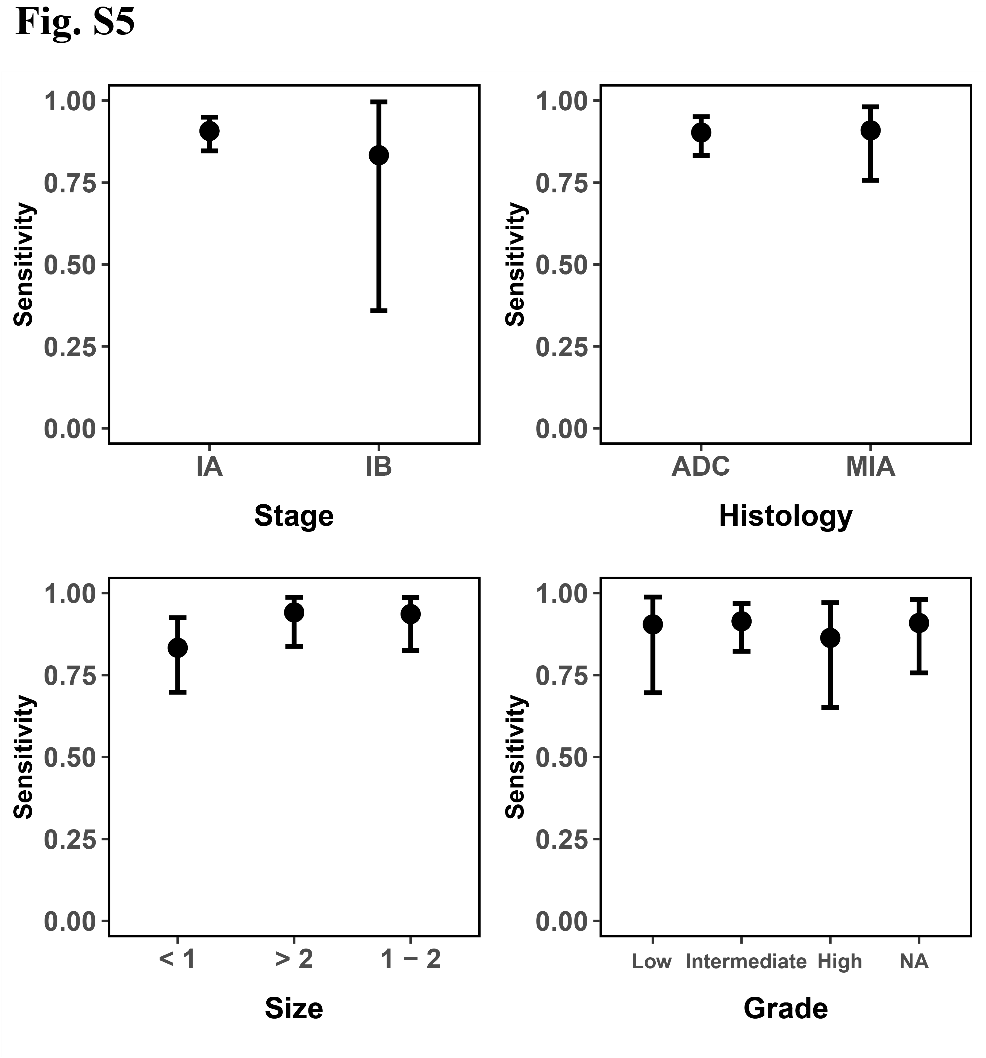


**Supplementary Figure 6. Cancer origin scores of the false-negative samples.** PLC: Primary liver cancer; CRC: Colorectal cancer; LUAD: lung adenocarcinoma.


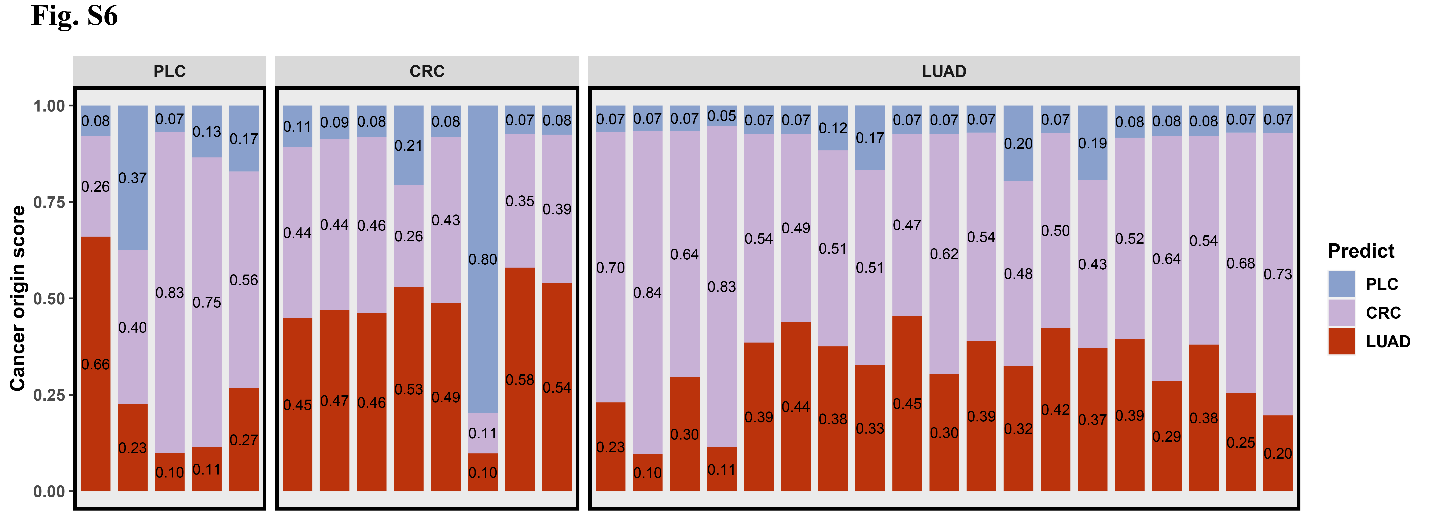

Supplement: Supplementary file 2 — Additional file 2: Supplementary Materials and Methods. Supplementary Figure 1. Distribution of cancer scores by cancer stages. Supplementary Figure 2. Cancer detection model evaluation using matched test cohort and cross-validated training cohort. Supplementary Figure 3. Evaluating cancer detection model sensitivity within different primary liver cancer subgroups. Supplementary Figure 4. Evaluating cancer detection model sensitivity within different colorectal cancer subgroups. Supplementary Figure 5. Evaluating cancer detection model sensitivity within different lung adenocarcinoma subgroups. Supplementary Figure 6. Cancer origin scores of the false-negative samples. [file 12943_2022_1594_MOESM2_ESM.docx]
